# Biological Interactions and Simulated Climate Change Modulates the Ecophysiological Performance of *Colobanthus quitensis* in the Antarctic Ecosystem

**DOI:** 10.1371/journal.pone.0164844

**Published:** 2016-10-24

**Authors:** Cristian Torres-Díaz, Jorge Gallardo-Cerda, Paris Lavin, Rómulo Oses, Fernando Carrasco-Urra, Cristian Atala, Ian S. Acuña-Rodríguez, Peter Convey, Marco A. Molina-Montenegro

**Affiliations:** 1 Laboratorio de Genómica y Biodiversidad (LGB), Departamento de Ciencias Básicas, Universidad del Bío-Bío, Chillán, Chile; 2 Laboratorio de Complejidad Microbiana y Ecología Funcional, Instituto Antofagasta, Universidad de Antofagasta, Antofagasta, Chile; 3 Centro de Estudios Avanzados en Zonas Áridas (CEAZA), Facultad de Ciencias del Mar, Universidad Católica del Norte, Coquimbo, Chile; 4 Departamento de Botánica, Facultad de Ciencias Naturales & Oceanográficas, Universidad de Concepción, Concepción, Chile; 5 Laboratorio de Anatomía y Ecología Funcional de Plantas (AEF), Instituto de Biología, Facultad de Ciencias, Pontificia Universidad Católica de Valparaíso, Valparaíso, Chile; 6 Centro de Ecología Molecular y Aplicaciones Evolutivas en Agroecosistemas (CEM), Instituto de Ciencias Biológicas, Universidad de Talca, Talca, Chile; 7 British Antarctic Survey, NERC, High Cross, Cambridge, United Kingdom; 8 Research Program "Adaptation of Agriculture to Climate Change" PIEI A2C2, Universidad de Talca, Talca, Chile; McGill University, CANADA

## Abstract

Most climate and environmental change models predict significant increases in temperature and precipitation by the end of the 21^st^ Century, for which the current functional output of certain symbioses may also be altered. In this context we address the following questions: 1) How the expected changes in abiotic factors (temperature, and water) differentially affect the ecophysiological performance of the plant *Colobanthus quitensis*? and 2) Will this environmental change indirectly affect *C*. *quitensis* photochemical performance and biomass accumulation by modifying its association with fungal endophytes? Plants of *C*. *quitensis* from King George Island in the South Shetland archipelago (62°09′ S), and Lagotellerie Island in the Antarctic Peninsula (65°53′ S) were put under simulated abiotic conditions in growth chambers following predictive models of global climate change (GCC). The indirect effect of GCC on the interaction between *C*. *quitensis* and fungal endophytes was assessed in a field experiment carried out in the Antarctica, in which we eliminated endophytes under contemporary conditions and applied experimental watering to simulate increased precipitation input. We measured four proxies of plant performance. First, we found that warming (+W) significantly increased plant performance, however its effect tended to be less than watering (+W) and combined warming and watering (+T°+W). Second, the presence of fungal endophytes improved plant performance, and its effect was significantly decreased under experimental watering. Our results indicate that both biotic and abiotic factors affect ecophysiological performance, and the directions of these influences will change with climate change. Our findings provide valuable information that will help to predict future population spread and evolution through using ecological niche models under different climatic scenarios.

## Introduction

The Antarctic continent is among the most stressful environments on Earth for plant life [1–3], with their establishment and survival limited by conditions such as low temperatures, desiccation, wind abrasion, high radiation, and low water and nutrient availability [4–6]. Over the last several decades, the Antarctic Peninsula has been the most rapidly warming region of the Southern Hemisphere [7–11]. By the end of the 21st Century, climate change models [12] predict significant increases in global mean annual temperatures (2.8°C, ranging from 1.6 to 5°C) and precipitation (20–25%, ranging from -2% to +35%). Although global climate change (GCC) is expected to have profound impacts on ecosystems worldwide, through the processes of ‘polar amplification’ high latitude ecosystems–including those of the Arctic and Antarctic–will both experience greater magnitudes of change for which are more sensitive than others to these changes [12–14]. As chronically low temperatures and low water availabilities currently characterize Antarctic terrestrial ecosystems [15–17, 2], climate change is expected to have positive impacts on Antarctic plant growth and survival [6, 18–19]. However, experimental evidence from studies of Arctic plant species has so far been equivocal, with warming having positive [20–21], neutral or negative effects [22–23], on plant photosynthetic rate, vegetative growth, and reproductive output.

Globally, a wealth of studies has addressed the impact of some of the component of global environmental change on vegetation [e.g., 10–11, 13–14, 21–22], but there is a dearth of data on the impacts of multiple factors and in particular their interactions. Plant responses to multiple factors may be (1) additive, when there is no interaction between responses, (2) synergistic, when the outcome is greater than the additive impacts of the individual component, and (3) antagonistic, when some effects counteract others. Experimental approaches that include multivariate interactions are thus appropriate and likely to generate ecologically relevant information.

Abiotic interactions are not alone in modulating plant performance. A large number or studies have shown that biotic interactions such as competition and herbivory can have significant effects on plant performance [24–25]. Furthermore, the impacts of biotic interactions can change along environmental gradients. Fungal endophytes are ubiquitous plant symbionts that can strongly influence plant physiological performance, particularly under stressful conditions [26]. They can confer fitness benefits to host plants including tolerance to herbivory, heat, salt, disease and drought, amongst other stress factors (e.g. [27–30]). However, despite their known potential to drive the ability of plants to cope with stressful environmental conditions, little is known about how climate change might affect plant performance through modifications of symbiotic interactions (but see [26]). Symbiotic interactions between fungi and higher plants and liverworts in the Antarctic environment have been demonstrated [31–34]. Recent reports have focused on the occurrence, type of association, diversity and possible ecological roles of mycorrhizal and dark septate endophytic fungal interactions with vascular plants. Only two native vascular plants, both occurring on the Antarctic Peninsula, are exposed to the extreme environmental conditions of the Antarctic [4, 35]. Upson et al. [32, 36] and Rosa et al. [33–34] described the root-fungal associations of both plants along a latitudinal transect in the Antarctic, demonstrating that root endophytes were able to mineralize peptides and amino acids in the rhizosphere, increasing nitrogen availability to the roots of plants. Further study is clearly required to understand the potential role of these positive interactions in the adaptation of plants to environmental stresses and changes in the Antarctic ecosystem. Increased understanding of the role of root endophytes in the establishment process and subsequent survival is also paramount for understanding and predicting the future spread of these vascular plants under current climate change scenarios.

The two vascular plants (*Colobanthus quitensis* and *Deschampsia antarctica*) occurring naturally on the Antarctic continent [4, 37] have their southern limit of distribution in the Antarctic Peninsula. *C*. *quitensis* (Kunth) Barttl. (Caryophyllaceae), commonly known as the Antarctic pearlwort, is a small-sized cushion-like perennial herb, self-compatible in sexual reproduction, however vegetative reproduction is the more common means of propagation in this species in Antarctica [15, 38]. This species is characterized by an extremely wide distributional range spanning from Mexico (17°N) to the southern Antarctic Peninsula (69°S) [39–40]. This wide distribution has been attributed to the high levels of phenotypic plasticity and ecotypic differentiation in both morphological and physiological attributes [19, 41–42]. Furthermore, since abiotic Antarctic environmental conditions are expected to become more benign for plant life due to GCC, a reduction in the positive net effects of microorganisms (e.g., root-fungal endophytes) on the physiological performance of *C*. *quitensis* can be predicted under a GCC scenario.

The main goal of this study was to determine the direct (through modifications of temperature and water availability) and indirect (through modifications of the interaction between the plant and its associated fungal endophytes) effects on the performance of *C*. *quitensis*. We specifically addressed the following questions: (1) Do abiotic factors (temperature and water) differentially affect the photochemical performance of *C*. *quitensis*?, and (2) Will GCC indirectly affect *C*. *quitensis* photochemical performance and biomass accumulation by modifying its association with fungal endophytes? We predicted that: (i) GCC will have positive direct effects on the photochemical performance and fitness of *C*. *quitensis* and (ii) GCC will indirectly reduce the importance of the symbiotic interaction, without affecting to *C*. *quitensis*.

## Methods

### Plant sampling and study sites

Individuals of *Colobanthus quitensis* were collected in two Antarctic sites: close to the Polish Antarctic Station “Henryk Arctowski”, King George Island, South Shetland Islands (62°09′ S), and Lagotellerie Island in the Antarctic Peninsula (65°53′ S). All plants (between 5 and 15 cm diameter) were collected during the 2011–2012 growing season. Each individual plant was excavated together with the soil around the roots (ca. 250 g) and kept well-watered in a plastic box under natural conditions of light and temperature for 1 h until the transplant experiment in situ or for its transportation to be used in growth chambers experiments at the Centro de Estudios Avanzados en Zonas Áridas (CEAZA), La Serena, Chile (29°54′ S). Plants transported to CEAZA were replicated by vegetative propagation in order to increase the biological material for manipulative experiments conducted in the growth chambers. Plants used in the experiments with controlled condition (CEAZA) were generated from an initial pool of plants collected in the field between 1 and 100 m from each other, in the hopes of obtain greater genetic variability. Although sexual reproduction in limited in *C*. *quitensis* [15–38], it maintains low but significant levels of within-populations genetic diversity [43].

To conduct all manipulative experiments in field as well as collect of individuals to be used in chamber experiments we counted with international permits and authorizations given by the Chilean Antarctic Institute. Additionally, we confirm that all these studies not involve endangered species.

### Climate change experiments

We designed two experiments to assess how Global Climate Change (GCC) modulates the effects of abiotic and biotic variables on the ecophysiological performance of *C*. *quitensis*. The consequences of the changes in current and future environmental conditions on plant photochemical performance were examined in growth chambers with an automatic system of air cooling (Model: LTJ300LY; Tianyi Cool, China), while the effects of abiotic variation were evaluated through a field experiment. In order to establish the “current environmental conditions” (control treatment) for these, we used both published and contemporary field data. These data were then used to define the “future environmental conditions” treatment, through modifying the current conditions based on the most recent global change predictions and models [9, 12]. First, we selected a temperature of 4°C as control (current environmental temperature). This temperature was chosen because it represents an intermediate value between the King George Island (5°C) and the Antarctic Peninsula (3°C, www.worldclime.org) over their growing seasons (from December to February). Current global and regional climate models predict average annual temperature increases of 2–4.5°C [12, 44] over the next century in Antarctica [9, 11]. Thus, incubation temperature was increased by 3°C in our “future conditions” experiments.

Two growing chambers (Forma Scientific, Inc) were used for these experiments, one set at 4°C (“current climatic conditions treatment”) and other at 7°C (“warming treatment”; hereafter +T°). Both chambers were operated with a photosynthetically active photon flux density (PPFD) of 150 μmol m-2 s-1 and an 18/6 h light/dark period. The light source was provided by F40CW cool-white fluorescent tubes (General Electric). Since the effects of a treatment can be affected by the characteristics of the growth chamber (see [44, 45]), different treatments were conducted in both chambers and individuals were transferred between chambers, changing the settings in the abiotic conditions. Plants were maintained in plastic pots (300 cc) filled with native soil (soil around plants obtained from every site). Plastic pots positions were randomized within the growth chambers every week. At the end of each month, the chambers were switched off, cleaned, and individuals were transferred between chambers, and growth conditions were re-established [45].

Finally, in order to establish the irrigation levels for control and water addition treatments (+W, below), we measured water availability in a natural *C*. *quitensis* population located in the vicinity of the Polish Antarctic Station, South Shetland Islands (62°39`S 60°36`W). At this site, soil matrix potential was measured 10 cm from each of 12 randomly chosen individuals using a tensiometer Jet Fill 2725 Series (Soil Moisture, Co, USA). Measurements were conducted in two consecutive growing seasons (2008–09 and 2009–10). Tensiometers were buried 20 cm deep and matrix potential was recorded after a 30 min stabilization period. All measurements were made between 12:00 and 14:00 h, on four consecutive days and the data merged. As both growing seasons showed similar water availability results the two sets of data were merged (data not shown). Given that most climate models have predicted increases of water availability of 15–20% [12, 46], we used an increase of 20% in water availability for the future conditions experiments. To achieve field-measured values (-24 kPa) under experimental conditions, plants were irrigated every 5 d with 70 ml of water. Thus, the +W treatment consisted in irrigating plants every 5 d with 84 ml of water.

We evaluated the effect of GCC on *C*. *quitensis* performance by measuring three response variables that are positively correlated with overall fitness. First, we estimated photochemical performance of PSII as the maximum quantum yield of PSII (Fv/Fm; where Fv = [Fm–F0], Fm = maximum fluorescence yield, and F0 = minimum fluorescence yield) using a pulse modulated-amplitude fluorimeter (FMS 2, Hansatech, Instrument Ltd, and Norfolk, UK). The measure Fv/Fm has been correlated with plant fitness and is a very good approach to the state of health of the photosynthetic system of a plant [47]. A group of leaves from each individual was dark-adapted for 30 min (to obtain open PSII centers) using a black-box (30 x 20 x 15 cm) to ensure maximum photochemical efficiency. Second, the direct effects of GCC on *C*. *quitensis* were evaluated in terms of the mean total biomass. After the end of the experiments, whole plants were harvested (root plus shoot), oven-dried for 48 h at 70°C and individually weighed using a digital balance. The third fitness-related variable was the percentage of plants generating flowering structures. This was documented before the end of the experiments (after 4 months of growth).

### Experiment 1: Evaluating the direct effects of the increase in water availability and warming in *C*. *quitensis* fitness related traits

We evaluated the effects of watering and warming on *C*. *quitensis* fitness-related traits in two sites: King George Island and Antarctic Peninsula. A total of 48 plants per population were assigned to four treatments: warming (+T°, *n* = 12), watering (+W, *n* = 12), combined warming and watering (+T° +W, *n* = 12) and current environmental conditions (control: normal levels of water and temperature, *n* = 12). This experiment was performed in growth chambers and lasted 16 weeks to simulate the natural duration of the growing season. We measured three fitness-related traits as response variables: photochemical performance, total plant biomass, and reproductive effort (measured as flowering percentage). As describe above, all individuals were re-arranged every week to minimize any chamber-specific effects.

### Experiment 2: Evaluating the effects of global change on *C*. *quitensis* through indirect impacts on its associated fungal endophytes

A field experiment was performed during the 2011–2012 growing season in the vicinity of the Polish Antarctic Station, King George Island in order to evaluate if GCC can indirectly effect *C*. *quitensis* fitness-related traits through modifying the biotic interaction between *C*. *quitensis* and its associated root-endophytic fungi. In this case, the ‘future’ scenario was simulated in a manipulation placed in the field through an increase in water availability (+W; 20% increase in water addition). A total of 60 *C*. *quitensis* individuals were assigned either to a water addition (+W) treatment (*n* = 30) or to a current moisture treatment (*n* = 30). Within each treatment, the 30 plants were randomly assigned to three further treatments: i) plants growing in pots filled with non-sterilized native soil (hereafter NS), ii) plants growing in pots filled with sterilized native soil (hereafter S-NS), and iii) plants growing in pots filled with sterilized native soil, re-inoculated with fungal endophytes (hereafter RI-SNS). Plants were grown in a sterilized mixture of rhizospheric soil (taken from the study site), perlite and sand (1:1:1), preventing initial contamination with other microorganisms. The inoculation procedure used a single strain of fungal endophyte isolated from *C*. *quitensis*, which was selected since its higher frequency in the host plant. This strain was previously identified as *Penicilluium chrysogenum* using ITS and 28S sequencing [48] (Genbank code: KJ881371). The endophyte was cultured on PDA medium diluted by a factor of ten, supplemented with 50–100 mg/ml of ampicillin, tetracycline, and streptomycin, and cultures were then grown at 22°C under a 12/12 h light/dark regime. After 5–14 days of growth, conidia were harvested from plates by adding 10 ml of sterile water and gently scraping off spores with a sterile glass slide. The final volume of spores was adjusted to 100 ml with sterile water, filtered through four layers of sterile cotton cheesecloth gauze, and spore concentration adjusted to 10^6^–10^7^ spores/ml. Lastly, plants were inoculated and grown under growth chamber conditions described above previous transplant in field in the vicinity of Arctowski station, King George island. The inoculation was repeated three times to ensure fungal association, and verification of an effective symbiosis was evidenced by microscopy. On the other hand, to obtain endophyte-free plants from clonal material, the commercially available fungicide “Benlate” (DuPont, Wilmington, DE, USA) were used. Benlate was chosen as no phytotoxic effects have been detected on perennial ryegrass [49]. Leaves and roots were completely submersed in tap water containing 2 gl^–1^ of Benlate and maintained for 1 h at room temperature. After 4–5 weeks of growth, newly emerged tillers were examined for the presence of fungal infection. Assessing endophyte colonization was performed on a subset of at least 10% of total plants. Before the beginning of the experiment, two plants per treatments were sacrificed to check microscopically for the presence and/or absence of endophytes by routine staining. Three response variables were measured in *C*. *quitensis* plants assigned to the three experimental treatments: photochemical performance (*F*_v_/*F*_m_), total biomass and adult survivorship. The first two response variables were measured as described above. The survival of transplanted plants in the field was recorded every week over one month of experimental period.

### Statistical analyses

In Experiment 1, the effect of site (King George Island and Antarctic Peninsula) and treatments (+T°, +W, +T°+W, and control) on the photochemical performance (*F*_v_/*F*_m_) and total biomass of plants from King George Island and Antarctic Peninsula were analyzed using factorial ANOVAs and Tukey HSD (α = 0.05) as an *a posteriori* test. In Experiment 2, the survival curves of *C*. *quitensis* grown in native, sterilized and re-inoculated native soils were compared using the Kaplan-Meier method. The statistical significances of the different survival curves were estimated using Cox-Mantel tests (Fox 1993). The differences in photochemical performance of PSII (*F*_v_/*F*_m_) and plant biomass were compared using factorial ANOVA. For all the ANOVAs, the assumptions of normality and homogeneity of variances were evaluated using Shapiro-Wilks and Bartlett tests, respectively [50]. In all cases *post hoc* comparisons were made using Tukey HSD tests. All analyses were performed with Statistica 6.0.

## Results

### Experiment 1: Evaluating the direct effects of increase in water availability and warming on *C*. *quitensis* fitness-related traits

**Fig 1:**
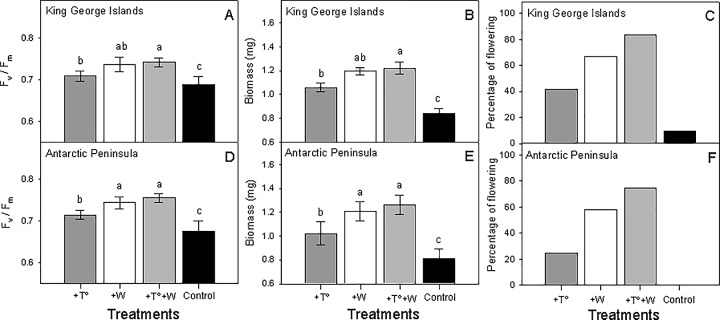
Results of global climate change simulation experiments. Photochemical performance, measured as photochemical efficiency (*F*_v_/*F*_m_) of photosystem II (PSII), and total biomass and the percentage of flowering plants from King George Island (A, B, C) and Antarctic Peninsula (D, E, F) are shown. Plants from all origins were exposed to four experimental treatments (temperature increase, water availability increase, temperature plus water increase and control). Bars are means (± SD). Bars labeled with different lowercase letters are significantly different (Tukey HSD tests, α = 0.05).

The factorial ANOVA revealed that site did not affect photochemical efficiency and biomass (Table 1). There was a significant interaction between site and treatments for photochemical efficiency (Table 1), indicating that the simulated climatic conditions will have differential effects on different sites. Plants from King George Island exposed to simulated warming showed a significant increase in photochemical performance (Fig 1A), biomass (Fig 1B) and percentage of plants flowering (Fig 1C). Similarly, the +W treatment increased the photochemical performance (Fig 1A) and biomass (Fig 1B) with respect to control plants. The effect of the combined +T°+W treatment on photochemical performance was significantly greater than that of the +T° treatment alone, but similar to that of the +W treatment, suggesting that +W has stronger effects than +T° (Fig 1A). Plants from the Antarctic Peninsula also increased their photochemical performance (Fig 1D), biomass (Fig 1E) and percentage of plants flowering (Fig 1F) in response to +T° and +W. As with King George Island plants, the interaction treatment (+T°+W) had stronger effects on photochemical performance (Fig 1D), biomass (Fig 1E) and percentage of plants flowering (Fig 1F) compared to the +T° treatment.

**Table 1:** Results of the factorial ANOVAs evaluating the interactive effects of the site (King George Island and Antarctic Peninsula) and treatments (warming (+T°), watering (+W), simultaneous warming and watering (+T°+W) and controls) on (A) photochemical performance, measured as maximum quantum yield (*F*_v_/*F*_m_) of photosystem II (PSII), and (B) total biomass of *Colobanthus quitensis*. Significant *P* values (< 0.05) are highlighted in bold.

Source of variation	d.f.	MS	*F*	*P*
**(A) Photochemical efficiency (*F***_**v**_**/*F***_**m**_**)**				
Site (S)	1	0.0003	1.30	0.266
Treatments (T)	3	0.0217	87.40	**< 0.0001**
S x T	3	0.0008	3.30	**0.002**
Error	88	0.0003	-	-
**(B) Biomass (gr)**				
Site (S)	1	0.001	0.01	0.936
Treatments (T)	3	0.8372	61.84	**< 0.0001**
S x T	3	0.0114	0.84	0.473
Error	88	0.0135	-	**-**

### Experiment 2: Evaluating the effects of environmental change on *C*. *quitensis* through indirect impacts on its associated fungal endophytes

**Fig 2:**
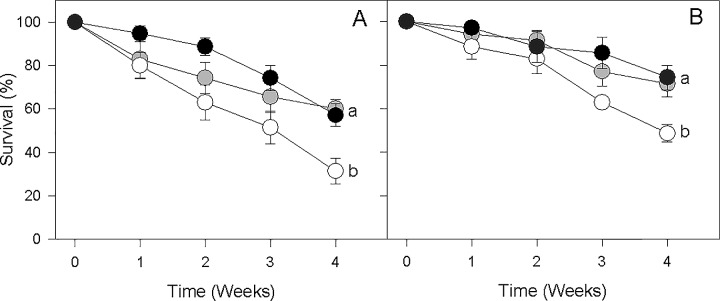
Survival of *C. quitensis* grown in three different soil types: non-sterilized native soil (NS, grey circles), sterilized native soil (S-NS, white circles), and sterilized native soil, re-inoculated with fungal endophytes (RI-SNS, black circles) under current (A) and simulated climatic change scenarios (B). Circles are means (± SE). Different letters indicate significant differences (Cox-Mantel tests, α = 0.05).

**Fig 3:**
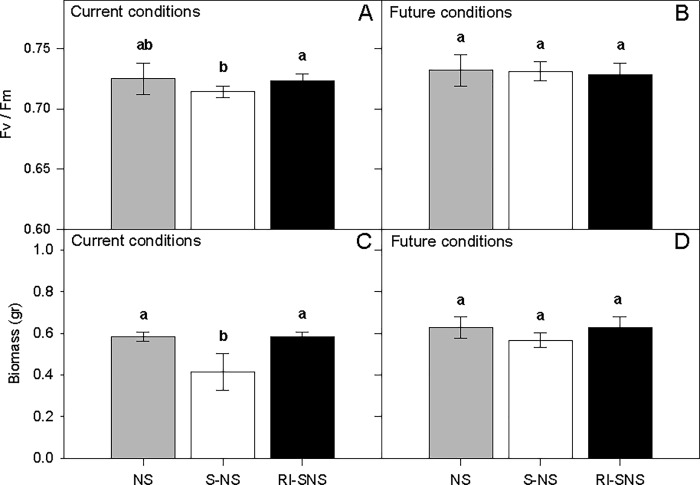
Photochemical performance, measured as maximum quantum yield (*F*_v_/*F*_m_) of photosystem II (PSII), and total biomass of *C*. *quitensis* growing in three different soil types (NS: non-sterilized native soil, S-NS: sterilized native soil, and RI-SNS: re-inoculated native soil) under current (A and C), and future climatic conditions (B and D; 20% increased water addition). Bars are means (± SD). Bars labeled with different letters indicate significant differences (Tukey HSD tests, α = 0.05).

The survivorship of *C*. *quitensis* grown in native or re-inoculated soils was significantly higher than in sterilized soil (i.e. without endophytes) under both contemporary and simulated future increased moisture conditions (Cox-Mantel Test *P* < 0.05, Fig 2A and 2B). The presence of fungal endophytes was also correlated with increased photochemical performance and biomass (Fig 3). Factorial ANOVA revealed that water addition led to significant increases in photochemical performance (*F*_1, 66_ = 18.8, *P* < 0.001; Table 2, Fig 3A and 3B) and biomass (*F*_1, 66_ = 45.17, *P* < 0.001; Table 2, Fig 3C and 3D). The interaction between water addition and soil treatments was only significant for plant biomass suggesting that, under wetter conditions, the presence of fungal endophytes will not increase plant growth (*F*_2, 66_ = 9.20, *P* = 0.001; Table 2, Fig 3C and 3D).

**Table 2:** Results of factorial ANOVA evaluating the interactive effect of water addition (+W: future conditions) and treatments (non-sterilized, sterilized and re-inoculated sterilized soils) on (A) photochemical performance, measured as maximum quantum efficiency (*F*_v_/*F*_m_) of photosystem II (PSII), and (B) the total biomass of *Colobanthus quitensis*. Significant *P* values (< 0.05) are highlighted in bold.

Source of variation	d.f.	MS	*F*	*P*
**(A) Photochemical efficiency (*F***_**v**_**/*F***_**m**_**)**				
Water addition (+W)	1	0.001	18.80	< **0.001**
Treatments (T)	2	0.002	2.77	0.069
+W x T	2	0.001	2.38	0.100
Error	66	-	-	-
**(B) Biomass (gr)**				
Water addition (+W)	1	0.114	45.17	< **0.001**
Treatments (T)	2	0.211	41.63	< **0.001**
+W x T	2	0.047	9.20	< **0.001**
Error	66	-	-	**-**

## Discussion

Our results indicate that experimentally simulated GCC has profound effects on the photochemical performance and fitness-related traits of *Colobanthus quitensis*, both through the consequences of direct modifications of abiotic conditions and the indirect modification of biotic interactions with endophytic fungi. Based on those results, shifts range after local scale population expansions in response to global change can be projected for *C*. *quitensis* for the next decades. These findings are key to understand how Antarctic plant communities will respond to future climatic conditions.

### Direct effects of simulated environmental change

Experimental increases in water availability, and the combination of water and temperature, had a stronger positive effect on *C*. *quitensis* photochemical performance than temperature alone. This suggests that there is a hierarchical response in the threshold to each abiotic factor, with changes in water being more responsive than temperature for photochemical performance.

Any realistic simulated global change scenario should not only consider warming but also parallel changes in other abiotic factors such as water [5]. It is well known that water stress produces changes in foliar attributes [51–52], reductions in photochemical performance [53–54] and reductions in reproductive effort [55–56]. Xiong et al. [57–58] showed that *C*. *quintensis* plants grown at an experimental temperature of 20°C produced 2.3 and 3.3 times more biomass and leaf area than plants grown at 7°C, respectively. They also showed that the optimal photosynthetic temperature of the *C*. *quitensis* plants from a site near Palmer Station (64°46' S; 64°00' W) was 13°C, while they could maintain around 30% of their maximum photosynthetic rate at 0°C. This indicates that low temperatures frequently limit photosynthesis in *C*. *quitensis* and also suggests that future warming will improve aboveground biomass allocation to leaf production.

Together with range shifts [59], changes in plant abundance [60–61] and phenology [62–63] provide overwhelming evidence that plants are responding to climate change. Over the past 50 years Antarctic Peninsula has warmed almost 1.3°C (Vaughan et al. 2003 [7]). The increases in size, reproduction and range shifts described for populations *C*. *quitensis* along Maritime Antarctica, over the last 25 years [18, 64–66], have been attributed to global warming. More recently, Cannone et al. [67] estimated that *C*. *quitensis* increased its coverage and number of colonized sites by 208% and 35%, respectively, over a period of 50 years. Accordingly, our findings support the notion that *C*. *quitensis* has increased its abundance as a consequence of past global warming, and also suggest that future warming will promote changes in phenology, abundance and southward range expansion.

It is important to acknowledge the limitations of our experimental design. Natural environmental conditions are virtually impossible to reproduce in growth chambers. Hence, our estimations of plant performance might differ from those we would find in the field, either because other biotic and/or abiotic variables were not measured. Thus natural conditions were not fully mimicked in our experimental setup and further field research is needed under field conditions.

### Indirect effects of simulated environmental change

The presence of endophytic fungi–both in natural soils and after re-inoculation of sterilized soils—had significant positive effects on *C*. *quitensis* growth, photochemical performance, and survival under both current and simulated future conditions. Their positive influence was stronger under current conditions than under the simulated future (assumed to be less stressful) conditions. It has been suggested that the positive symbiotic association between plant and fungal endophytes could be a key factor in the adaptation and performance of plant species, especially in stressful environments [68]. Previous studies in Antarctica have shown that the symbiotic association between fungal endophytes (dark septate fungi) and vascular plants is a general phenomenon [32–34] and is important in the plants’ success, possibly through their role in the uptake of nutrients and water [36, 69]. Functional symbiosis between Antarctic plants and endophytes appears to be an important strategy adopted by plants in order to survive the extreme environmental conditions of Antarctica. The stress gradient hypothesis proposes that, as stress levels increase, mutually supportive interactions became more significant (*sensu* [70]). However, a reduction in the significance of these interactions can be predicted for the maritime Antarctic ecosystems studied here in a GCC scenario, as stress will generally be reduced by increasing temperatures and water availability [6]. Our results are consistent with this hypothesis, as the positive effects of the presence of endophytic fungi were lower under less stressful conditions. Future studies should address the possible evolutionary consequences of environmental change on mutualistic relationships such as functional symbiosis between fungal endophytes and vascular plants.

### Concluding remarks and future perspectives

Nearly 15 year ago, Alberdi et al. [4] studied the ecophysiological mechanisms deployed by the only two native vascular plants -*C*. *quitensis* and *D*. *antarctica*- to cope with the antarctic environment. Some of these mechanisms were related to high freezing resistance and high photosynthetic capacity at low temperature. Recently, Cavieres et al. [35]) reviewed the mechanisms and adaptations deployed by antarctic vascular plants. Although both studies highlight the physiological capacity of these vascular plants to cope with environment, none of them assessed the effects of the future climate change (including biological interactions) on the photochemical performance and/or fitness of Antarctic plants. Thus, our study helps to fill a gap in the current literature of the ecophysiology of vascular Antarctic flora, since we show how biotic and abiotic factors modify the responses of *C*. *quitensis*, driving its capacity to adapt and survive in that environment. In this context, this study provides valuable experimental evidence showing how Antarctic vascular plants (e.g., *C*. *quitensis*) will expand its regional distribution in Antarctica in future decades.

On the other hand, the environmental changes (biotic and abiotic) predicted for the Antarctic ecosystems for the next 100 years include the prediction that Antarctic ecosystems will become less isolated than they are at present [6, 9, 71]. Low species richness and thus relatively simple community structure make Antarctic ecosystems particularly likely to change in response to colonization by non-native species [72]. Therefore, the arrival of new colonizing species, either through natural dispersal or anthropogenic assistance, is likely to affect the distribution and abundance of *C*. *quitensis*. Recent studies have reported the presence and increasing distribution of alien invasive plants such as *Poa annua* in sub-Antarctic and Antarctic ecosystems, with the latter now present in both in the South Shetland Islands and the northern Antarctic Peninsula [72–73]. Since the projected biotic and abiotic environmental changes will affect both the native and introduced components of the Antarctic flora, future studies should also address the interactions between native and alien plants in the context of change, in order to help predict how the terrestrial Antarctic landscape will change over coming decades.
